# Supplementary material for: Electrically driven organic laser using integrated OLED pumping
Source: Nature. 2023 Sep 27;621(7980):746–52. doi: 10.1038/s41586-023-06488-5 (PMC10533406; doi:10.1038/s41586-023-06488-5)
Supplement: Supplementary file 1 — . [file 41586_2023_6488_MOESM1_ESM.pdf]

nature portfolio

https://doi.org/10.1038/s41586-023-06488-5

Supplementary information

Electrically driven organic laser using integrated OLED pumping

In the format provided by the authors and unedited

Supplementary Table 1|Summary of performance of reported two-terminal vertical organic semiconductor devices operating at high current density.

Ref. No.	τ[i] (ns)	S[ii] (cm^2)	λ[iii] (nm)	J[iv] (kA/cm^2)	R[v] (W/cm^2)	EQE (%)
54	5.0E+03	1.E-02	610 (EL[vi])	0.057	1.04 (Est.)	0.9
55	1.0E+03	4.E-03	590 (EL[56])	0.01	N.A.[vii]	N.A.
57	2.5E+02	5.E-02	540 (EL)	0.7	0.05 (Est.[viii])	0.003 (Est.)
58	DC	1.E-14	530 (EL)	10	N.A.	N.A.
59	2.5E+02	2.E-02	564 (EL[2])	0.6	2.3 (Est.)	0.18 (Est.)
60	1.0E+02	1.E-02	564 (EL[2])	0.6	1.9 (Est.)	0.14 (Est.)
61	2.5E+02	3.E-04	Not relevant	1.3	N.A.	N.A.
62	1.0E+04	8.E-05	630 (EL[63])	0.3	N.A.	N.A.
64	N.A.	N.A.	530 (Assumed)	0.5	11 (Est.)	0.91 (Est.)
65	1.0E+03	2.E-05	565 (EL[66])	0.51	3.47 (Est.)	0.29 (Est.)
28	7.0E+04	8.E-03	630 (EL[63])	0.04	0.12 (Est.)	0.15
67	DC	2.E-05	Not relevant	1.1	N.A.	N.A.
68	5.0E+03	2.E-03	482 (PL[ix] [69])	0.093	7.1 (Est.)	3.0 (Est.)
70	5.0E+03	8.E-05	528 (EL[71])	0.57	0.9 (Est.)	0.07
72	5.0E+03	8.E-05	470 (PL)	0.56	N.A.	N.A.
73	DC	2.E-05	Not relevant	12	N.A.	N.A.
74	N.A.	1.6E-03	512	N.A.	16	N.A.
75	DC	6.E-06	550 (EL)	1.1	5.0 (Est.)	0.2
76	DC	4.E-10	Not relevant	6400	N.A.	N.A.
77	N.A.	1.E-02	600	0.028	0.23 (Est.)	0.4
78	2.0E+03	N.A.	Not relevant	0.03	N.A.	N.A.
79	5.0E+03	6.E-06	480 (PL[80])	0.1	2.2 (Est.)	0.85
81	5.0E+01	1.E-04	Not relevant	6.2	N.A.	N.A.
82	Quasi-DC	3.E-04	565 (EL[66])	0.07	N.A.	N.A.
83	DC	6.E-04	Not relevant	0.9	N.A.	N.A.
66	1.0E+04	NA	565	0.03	N.A.	N.A.
80	5.0E+03	3.E-04	480 (PL)	0.09	1.51	0.65

[i] τ is pulse width.
[ii] S is device active area.
[iii] λ is emission peak wavelength.
[iv] J is the current density.
[v] R is radiant exitance.
[vi] 'EL' represents the peak wavelength obtained from EL spectra
[vii] 'N.A.' indicates data which are not available from the reference
[viii] 'Est.' indicates values we have estimated based on the reported data
[ix] 'PL' represents the peak wavelength obtained from PL of emission layer.

**Ref. No.**	***τ*** **(ns)**	***S*** **(cm^2)**	***λ*** **(nm)**	***J*** **(kA/cm^2)**	***R*** **(W/cm^2)**	**EQE (%)**
84	5.0E+05	8.E-09	Not relevant	10	N.A.	N.A.
85	2.3E+01	N.A.	450 (EL[86])	0.12	2.5	0.76 (Est.)
87	5.0E+03	1.E-07	480 (PL[80])	2	5.2 (Est.)	0.1
32	5.0E+02	4.E-04	430 (EL[88])	0.553	4.31 (Est.)	0.27
89	5.0E+03	4.E-04	480 (PL[80])	0.27	5.93 (Est.)	0.85
90	5.0E+03	4.E-04	Not relevant	0.3	N.A.	N.A.
91	7.0E+01	1.E-04	Not relevant	5	N.A.	N.A.
92	3.0E+01	1.E-04	630 (EL[63])	3	12	0.2 (Est.)
93	2.5E+02	7.E-04	610 (EL[94])	0.4	N.A.	N.A.
95	9.0E+00	1.E-04	600 (EL[63])	2	N.A.	N.A.
96	1.0E+01	3.E-03	580 (EL)	3	0.42 (Est.)	0.0066 (Est.)
97	NA	4.E-04	480 (PL[80])	0.62	13 (Est.)	0.82
25	4.0E+02	1.E-05	480 (PL[80])	2	5.2 (Est.)	0.1
98	1.0E+01	2.E-04	600 (EL[63])	1.7	9.5	0.28 (Est)
31	4.0E+02	NA	480 (PL[80])	4.5	23 (Est.)	0.2
99	3.0E+02	1.E-03	550 (EL[100])	0.4	1.4 (Est)	0.15 (Est.)
101	4.0E+02	4.E-04	473 (PL)	0.61	N.A.	N.A.
102	1.0E+02	2.E-03	475 (PL)	0.1	0.13 (Est.)	0.05
103	2.5E+01	5.E-05	600 (EL[63])	8.8	N.A.	N.A.
104	4.0E+02	3.E-03	565(EL)	0.09	1.68 (Est.)	0.85
105	4.0E+03	3.E-03	510 (EL)	0.035	1.02 (Est.)	1.2
106	1.0E+00	1.E-04	528 (EL[71])	3	N.A.	N.A.
47	5.0E+03	8.E-03	430 (EL)	0.11	1.62 (Est.)	0.5
This work	5.4E+00	1.E-03	430	6.3	47	0.26
